# Unveiling the role of political education for political participation in China

**DOI:** 10.1016/j.heliyon.2024.e31258

**Published:** 2024-05-14

**Authors:** Mo Chen, Ghulam Rasool Madni

**Affiliations:** a School of Marxism, Yantai Vocational College, Yantai City, Shandong Province, 264000, China; b Department of Economics, Division of Management and Administrative Science, University of Education, Lahore, Pakistan

## Abstract

It is a well-known fact that political education plays a pivotal role in shaping informed, engaged, and responsible citizens in a country. The empirical literature lacks the exploration of the impact of political education on political participation in addition to political efficacy and social media usage. This study investigates the interconnected relationship between political education, social media usage, political efficacy, and political participation in China. Drawing upon a sample of 843 participants across various regions, this research explores the extent to which these factors influence political participation within the Chinese context by applying the structural equation modelling for empirical results and establishing a positive association between political education and political participation. Furthermore, the mediating role of social media and political efficacy is uncovered in relationship of political education and political participation in China. It is suggested to emphasize and invest in comprehensive political education programs through colleges, universities and social media that promote critical thinking, information literacy, and political responsibility for the well-being of the Chinese society.

## 1 Introduction

Man is by nature a political animal, according to the famous Greek philosopher, Aristotle. Politics is essential in shaping societies, influencing policies, and determining how communities function [1]. It provides the framework for governance, establishing laws, regulations, and institutions that manage a society. Political processes shape policies that address social, economic, and environmental issues. Education and politics are closely linked as education equips the individuals with critical thinking skills, knowledge of history, economics, and social sciences, enabling them to make informed decisions in the political sphere [2]. Informed citizens are essential for a healthy democracy as they can analyze policies, understand their implications, and participate actively in the democratic process. Education nurtures future political leaders by providing them with the necessary skills, knowledge, and understanding of societal issues [3]. It prepares individuals to become effective policymakers, administrators, and advocates for positive change. Political education is crucial for the well-being and development of a country [4]. It cultivates an informed and engaged citizens within a country. When people understand the political system, governance structures, and the impact of policies, they can make informed decisions during elections and actively participate in civic life [5].

Political participation is a two-way process that involves an active interaction between citizens and government [6]. In a political society, some individuals’ involvement in political processes and activities are autonomous, while in others it could be induced [7]. It encompasses a wide range of activities aimed at influencing government decisions, policies, or the overall direction of a political system [8]. This involvement can take various forms like voting, rallies, debates etc. Participation through voting in elections is most common form of political engagement [1,9]. It allows individuals to choose representatives, determine policies, and shape the leadership of their country or community [10]. Engaging in political campaigns, whether for a specific candidate, party, or cause, constitutes participation. This includes canvassing, organizing rallies, phone banking, and using social media to advocate for political change [11]. Becoming a member of a political party, interest group, or advocacy organization allows individuals to contribute to shaping policies, agendas, and political strategies [6]. Political participation is essential for the functioning of a democracy, as it ensures that the voice and concerns of a citizen is heard. It helps shape policies, holds leaders accountable, and contributes to a more inclusive and responsive political system [12].

Political education is fundamental for fostering and enhancing political participation through different ways. Political education empowers individuals by providing them with the knowledge and understanding of how political systems function [13,14]. It enhances awareness about the significance of civic engagement, voting rights, and the impact of political decisions on their lives and communities [15,16]. Secondly, it equips individuals with critical thinking skills to evaluate political information critically. They learn to discern between different sources of information, identify biases, and make informed judgments about political issues and candidates [11]. Thirdly, political education encourages active citizenship. When people are well-informed about political processes, policies, and the roles they play in a democracy, they are more likely to participate in voting, community organizing, and advocacy efforts [17]. Fourthly, an educated electorate comprehends the implications of various policies. This understanding enables individuals to assess how policies affect different societal groups, allowing for more informed and constructive participation in policy debates and decision-making processes [18]. Fifthly, a politically educated population is more likely to understand and appreciate diverse perspectives. This understanding fosters a more inclusive political environment where different voices are heard, and policies are crafted to reflect the needs of a diverse society [19].

Political efficacy is belief of an individual in its ability to understand and influence political affair. It's the confidence that the government and political institutions will respond to the demands and needs of the public [14]. It can be argued that high levels of political efficacy often lead to increased political participation. Individuals with a strong sense of efficacy are more motivated to participate in political activities [19]. They believe that their actions can make a difference, which encourages them to vote, join campaigns, participate in community initiatives, or engage in other forms of political activism [20].

Social media usage is another aspect that has significance for political participation. The relationship between political participation and social media usage has become increasingly intertwined in today's digital age [20]. Social media platforms have significantly influenced the landscape of political engagement and participation because it has lowered barriers to political participation by providing a platform for individuals to engage with political content, discussions, and campaigns from anywhere with an internet connection [21]. This accessibility has encouraged broader participation from a more diverse range of people [22]. Social media platforms serve as a rapid and widespread information-sharing tool. Users can access news, opinions, and diverse perspectives on political issues instantaneously. This dissemination of information helps users stay informed and engaged, which can drive political participation [23].

Existing literature on the relationship between social media use, political education, and political participation in China may fail to fully explain several key aspects of this relationship due to the unique characteristics of the Chinese political system and media environment. Earlier literature does not adequately address the impact of social media usage and political education in China. The Chinese government heavily regulates online content and monitors social media platforms, which can influence the types of political information available to users and their willingness to engage in political discussions. While existing studies may focus on formal political education, they may overlook alternative forms of political education, such as informal education through social networks, online forums, and other non-traditional channels. These alternative forms of education can play a significant role in shaping political attitudes and behaviors. While some studies may focus on the role of social media in political mobilization, they may not fully capture the diverse motivations behind social media use in China. Individuals may use social media for various reasons, including socializing, entertainment, and information seeking, which can influence their engagement with political content. These factors can shape individuals' access to information, their political attitudes, and their likelihood of engaging in political activities. Addressing these gaps in the literature could provide a more comprehensive understanding of the relationship between social media use, political education, and political participation in China and its implications for governance and political development.

This study fills several gaps in the existing literature by integrating the influence of political education, social media use, and political efficacy on political participation. By considering these factors together, the study provides a more comprehensive understanding of how they interact to influence political behavior in China. While existing literature may not fully explore the role of political education in shaping political participation in China, this study specifically examines the impact of political education on individuals' political attitudes and behaviors. This focus helps to highlight the importance of political education in fostering political engagement. Moreover, by investigating the mediating role of social media, the study contributes to understanding how social media use influences the relationship between political education and political participation. This aspect is particularly relevant in the context of China's strict control over the internet and social media platforms. In addition, this study also examines the mediating role of political efficacy, which is a key factor in determining individuals' willingness to participate in political activities. By considering how political efficacy mediates the relationship between political education, social media use, and political participation, the study provides insights into the psychological mechanisms underlying political behavior. This context-specific approach is important for understanding the dynamics of political participation in China.

The findings of this study contribute to existing theories in several ways. Civic empowerment theory posits that education and participation in civic activities empower individuals to participate in society and politics. The study's findings could support this theory by showing that political education enhances political efficacy, which in turn increases political participation. However, the study's focus on China's unique political context, including state control over education and media, could also challenge civic empowerment theory by highlighting the constraints on civic empowerment in authoritarian settings. Moreover, digital citizenship theory emphasizes the role of digital technologies, including social media, in promoting civic engagement and participation. The study's findings could support this theory by demonstrating that social media use mediates the relationship between political education and political participation in China. However, the study's focus on state-controlled social media and internet censorship could challenge digital citizenship theory by highlighting the limitations of digital technologies in promoting civic engagement in authoritarian contexts. In addition, social capital theory suggests that social networks and relationships facilitate collective action and political participation. The study's findings could support this theory by showing that social media use enhances political efficacy, which in turn increases political participation. However, the study's focus on the role of state-controlled social media in China could challenge social capital theory by raising questions about the nature of social networks and relationships in authoritarian settings and their impact on political participation.

The rationale of this study revolves around understanding the factors that influence political participation in China, with a focus on how political education, social media use, and political efficacy interplay in this context. Political education can play a crucial role in shaping individuals' political beliefs, attitudes, and behaviors. In China, where the government controls much of the education system, political education can significantly influence citizens' understanding of the political system, their level of political engagement, and their willingness to participate in political activities. Moreover, social media has become an increasingly important tool for political communication and mobilization worldwide, including in China. It provides a platform for citizens to access information, discuss political issues, and organize collective action. Understanding how social media use influences political participation, especially in the context of China's strict internet censorship and surveillance, is important for grasping the dynamics of political engagement in the country. In addition, high levels of political efficacy are associated with greater political participation, while low levels are linked to apathy and disengagement. By examining the mediating role of political efficacy, the study likely seeks to understand how individuals' beliefs about their own political efficacy influence the relationship between political education, social media use, and political participation in China.

After realizing the significance of political participation, this study has an objective to explore the impact of political education on political participation in China, in addition with exploring the role of political efficacy and social media usage in this relationship. Studying the role of political education, political efficacy, and social media usage for political participation in China is important for several reasons. China has a unique political system influenced by its history, culture, and government structures. Studying these factors in relation to political education, efficacy, and social media helps in comprehending the complexities of political participation within the country. China's educational system plays a significant role in shaping citizens' understanding of politics and governance. Studying the impact of political education can shed light on how ideological education and state narratives influence citizens' perspectives and participation. Political efficacy takes on a different dimension in China's authoritarian system [24,25]. Analyzing how citizens perceive their ability to influence political outcomes, engage with the government, and navigate the system provides insights into civic engagement within an authoritarian framework [26]. China has unique digital ecosystems with heavily controlled social media platforms. Understanding how social media usage influences political participation, despite censorship and state control, reveals strategies used by citizens to express dissent, engage in discussions, and mobilize for causes. Examining the relationship between these factors and political participation can offer insights into how Chinese citizens influence policy decisions, voice their concerns, and contribute to societal changes within the boundaries set by the government. Research on these aspects can lead to informed policy recommendations for enhancing civic education, improving political efficacy, and fostering meaningful political participation in China, which can contribute to societal development and governance reforms. Overall, studying the interplay between political education, efficacy, social media, and political participation in China provides valuable insights into the dynamics of political engagement within an authoritarian system, impacting both domestic and international perspectives on China's governance and society.

## 2 Theoretical relationship and research hypotheses

The relationship between political education and political participation is rooted in various theoretical perspectives that highlight how education influences and shapes individuals' engagement in the political process [27]. The civic education theory emphasizes the role of education in cultivating civic virtues, knowledge, and skills necessary for active citizenship [28]. Civic education aims to impart an understanding of democratic principles, rights, responsibilities, and the functioning of political institutions [29]. According to this theory, a well-rounded civic education can enhance individuals' understanding of their role in society, empowering them to participate more effectively in political activities [30]. On the other side, the political socialization theory focuses on how individuals acquire political beliefs, attitudes, and behaviors through social institutions, including education [31]. Political socialization within educational settings shapes individuals' perceptions of the political system, their sense of political efficacy, and their willingness to engage in political participation [32]. Schools and educational curricula play a significant role in shaping citizens' orientations toward politics and their likelihood of participating in political activities [33]. The resource mobilization theory is of view that education acts as a resource that empowers individuals to participate in politics. Higher levels of education are associated with increased access to information, critical thinking skills, and resources that enable individuals to engage more effectively in political processes [34]. Education provides individuals with the tools needed to navigate the complexities of the political system and effectively advocate for their interests [35]. Human capital theory views education as an investment in human capital. It posits that higher levels of education lead to greater political participation due to the skills, knowledge, and capabilities acquired. Individuals with higher educational attainment are more likely to engage in political activities as they perceive the benefits of their participation and possess the skills necessary for effective engagement [5,36]. Education that promotes democratic ideals, pluralism, and civic engagement contributes to the development of informed citizens who actively participate in the political process to uphold democratic principles [37]. Political education not only provides individuals with knowledge about politics but also instills values, skills, and attitudes that influence their willingness and ability to engage in political activities, thereby shaping the quality of democratic participation within a society. So it can be hypothesized as.

Hypothesis 1 Political education has a positive impact on political participation.

The relation between usage of social media and political participation encompasses various theoretical perspectives that shed light on how social media usage influences individuals' engagement in the political process. Network theory emphasizes the role of social networks and connections in facilitating political participation through social media [38]. Social media platforms act as channels that enable individuals to connect, share information, and mobilize support for political causes [10,39]. According to this theory, the structure and size of one's social network on these platforms influence their likelihood of engaging in political activities [12,40]. Information communication theory explains that social media serves as a powerful tool for disseminating political information and fostering political engagement. Information communication theory highlights how social media platforms facilitate the rapid and widespread dissemination of political content, news, and opinions [8,41]. These platforms enable users to access diverse sources of information, leading to increased political awareness and informed decision-making [4,15]. Selective exposure theory is of view that while social media provides access to a wide array of information, selective exposure theory suggests that individuals engage with content that aligns with their existing beliefs and preferences [6,42]. Consequently, the impact of social media on political participation might differ based on users' selective exposure [14]. Mobilization theory argues that social media plays a crucial role in mobilizing individuals for political action. Mobilization theory highlights how these platforms are used to organize and mobilize people for rallies, protests, campaigns, and other forms of collective action. Social media's capacity to rapidly disseminate calls for action and mobilize support can significantly impact political participation [17,19]. While social media has the potential to enhance political participation, digital divide theory suggests that unequal access to technology and digital literacy may exacerbate existing social inequalities, impacting individuals' ability to engage in online political activities [20,43]. Social media's influence on political engagement is shaped by factors such as network structures, information dissemination, mobilization efforts, selective exposure, digital access, and the dynamics of online interactions, all of which contribute to shaping individuals' participation in the political process [22,44].

Hypothesis 2 Social media usage has positive relationship with political participation.

The relation between political efficacy and political participation illustrate how individuals' beliefs about their ability to influence politics influence their engagement in the political process. Rational choice theory posits that individuals engage in political participation when they perceive that their actions will have a meaningful impact [45]. Political efficacy, in this context, influences participation as individuals weigh the costs and benefits of engaging in political activities based on their belief in their ability to affect political outcomes [16,46]. Civic voluntarism model describes that individuals participate in politics driven by their sense of duty and social responsibility. Political efficacy plays a crucial role as it influences an individual's sense of obligation to engage in political activities. Higher levels of efficacy might lead to increased participation due to a stronger sense of civic duty [25,47]. Mobilization theory argues that political efficacy is a key factor in mobilizing individuals for political action. When individuals have high levels of political efficacy—both internal (belief in their own abilities) and external (belief in the responsiveness of the political system)—they are more likely to engage in political participation, as they believe their actions will bring about desired outcomes [26,48,49]. Psychological empowerment theory emphasizes the psychological aspect of political efficacy [27]. Individuals with a strong sense of efficacy feel empowered and confident in their ability to engage in politics, which, in turn, motivates them to participate actively in the political process [28,50]. Individuals who believe they have the capacity to influence political outcomes and perceive the political system as responsive are engaged in political activities [51].

Hypothesis 3 Political efficacy has a positive relationship with political participation.

It is hypothesized in this study that social media usage acts as a mediator in relationship between political education and political participation. Social media serves as a platform for accessing and disseminating political information [52]. Political education might influence individuals to seek out political content, understand it better, and critically engage with it. This engagement with political information through social media might then prompt individuals to participate in political activities based on their increased knowledge and awareness [53]. Social media mediates the relationship by facilitating mobilization and engagement. Political education might enhance individuals' understanding of political issues and their efficacy in participating. Social media platforms then provide avenues for mobilization, encouraging individuals to actively participate in campaigns, rallies, or other forms of political engagement [54]. Selective exposure on social media could mediate the relationship. Individuals with higher political education might actively seek out diverse viewpoints and information. Social media, acting as a mediator, exposes them to a broader range of political perspectives and discussions, potentially influencing their subsequent participation in political activities [29,55]. Social media facilitates political discussions and deliberation. Individuals with higher political education might engage in more substantive and informed political discussions online. These discussions could serve as a mediator between political education and participation, as they might encourage individuals to translate their discussions into actual political participation [30,56]. In summary, social media usage can act as a mediator between political education and political participation by facilitating information access, mobilization, exposure to diverse perspectives, fostering political discussions, and enabling social mobilization campaigns [57]. These mediating pathways illustrate how social media plays a crucial role in influencing individuals' political engagement based on their level of political education.

Hypothesis 4 The social media usage has mediating role in relationship of political education and political participation.

Political education enhances individuals' knowledge, understanding of political processes, and critical thinking skills. This increased education could positively influence individuals' political efficacy by boosting their confidence in their ability to understand political issues and effectively participate in the political process [43,58]. Higher political efficacy, in turn, can motivate individuals to engage in political participation, believing that their actions can make a difference [59]. Individuals with higher political education might develop a stronger sense of internal political efficacy—belief in their own capabilities to influence politics—due to their increased knowledge base [23,60]. This higher internal efficacy might lead to increased political participation, as individuals with a stronger sense of efficacy are more likely to engage in various political activities, such as voting, campaigning, or joining political organizations [61,62]. Political education might influence individuals' perceptions of the political system's responsiveness to citizen input. Higher levels of political education could lead to a greater sense of external efficacy—belief in the responsiveness of the political system [31,63]. This belief that the system listens to and acts on citizens' voices can motivate individuals to participate more actively in politics, assuming that their efforts will yield results [32,64]. Political education might enhance individuals' ability to critically evaluate political information and make informed decisions. This increased competence can lead to higher political efficacy, as individuals feel more capable of understanding complex political issues and participating in political processes. This sense of competence and confidence can then motivate individuals to engage in political activities [34,65]. Higher political efficacy can also act as a motivational factor in mobilizing individuals for political participation. Individuals with stronger beliefs in their ability to influence politics (high efficacy) may be more inclined to participate actively, using their knowledge acquired through political education to inform their actions [33,66]. Political efficacy influences individuals' beliefs in their ability to understand politics, influence the system, make informed decisions, and be effective participants [67]. Higher political efficacy, fostered by political education, can serve as a crucial motivational factor in encouraging individuals to engage more actively in the political process.

Hypothesis 5 Political efficacy has mediating role in relationship of political education and political participation.

**Fig. 1:**
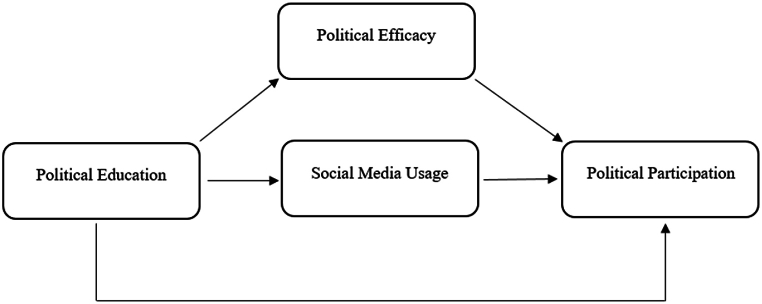
Conceptual framework.

The conceptual framework of the study is given in the following Fig. 1.

## 3 Materials and methods

To test the theoretical hypotheses, the data is collected from residents of China from June 1, 2023 to October 30, 2023. The questionnaire was sent via emails and social media. The convenience sampling method is used to select the participants of the study. Before sending the questionnaire to respondents, a written consent was obtained. The Likert scale is used to measure each item ranging 1–5 points, corresponding to five levels of "strongly disagree" to "strongly agree". To ensure the questionnaire's reliability, a preliminary survey of limited scale was carried out. Questions that were found to have unclear meanings or ambiguity were modified on the basis of feedback received during the preliminary survey. Respondents of age more than 18 years were selected. The response from under the age of 18 participants, is not considered for further empirical analysis of the study. In total, 1050 questionnaires were sent and 843 of them were suitable. The responses with missing values are excluded from the analysis. The basic information of respondents is given in the following Table 1.

**Table 1:** Descriptive analysis (N = 843).

Category	Item	Frequency	Proportion
Gender	Male	441	52.31
	Female	402	47.69
Marital Status	Married	432	51.24
	Single	411	48.76
Education	High School	121	14.35
	Bachelor	454	53.85
	Master	231	27.40
	PhD	37	4.39
Age	18–29 Years	416	49.35
	30–39 Years	301	35.71
	40–50 Years	103	12.22
	More than 50 Years	23	2.73
Residence	Urban Area	443	52.55
	Rural Area	400	47.45

### 3.1 Variables

#### 3.1.1 Political participation

One of main variables of this study is political participation. According to the Huntington and Nelson [68], “political participation is the activity of citizens trying to influence government decision-making.” There are 7 questions asked to measure the political participation adopted from “(1) voted or not ever since you became eligible to vote; (2) participated unit/village campaign meetings or rally; (3) mobilized others vote for a certain candidate; (4) expressed an opinion to local government leaders; (5) contacted other influential people outside the government; (6) got together with others to try to resolve local problems; (7) got together with others to raise an issue or sign a petition.”

#### 3.1.2 Political education

Political education is the process of imparting knowledge, understanding, and skills related to political systems, governance, civic duties, and political participation. It aims to educate individuals about political structures, processes, ideologies, and issues, fostering informed and engaged citizens within a society [69]. These questions are used to measure the political education of citizens. (1) How would you rate your understanding of your country's political structure and government institutions? (2) Can you name the ten branches of government and their respective roles? (3) Do you know about the electoral process in your country? (4) How familiar are you with the major political parties or movements in your country? (5) Are you aware of your ability to influence political decisions through means such as voting or activism? (6) How frequently do you engage in discussions about politics with friends, family, or colleagues? (7) Have you attended any political education workshops, seminars, or programs in the past year?

#### 3.1.3 Political efficacy

Political efficacy is an individual's belief in their ability to understand and influence political events and decision-making processes. To measure political efficacy, this study used the following items to measure political efficacy adopted from earlier studies [70,71]. “(1) I know more about politics than most people my age. (2) When political issues or problems are being discussed, I usually have something to say. (3) I consider myself well qualified to participate in politics. (4) I don't think public officials care much what people like me think. (5) The government cares a lot about what all of us think about new laws. (6) Sometimes politics and government seem so complicated that a person like me can't really understand what is going on; (7) People like me don't have any influence over what the government does” [71].

#### 3.1.4 Social media usage

Social media usage is the extent and manner in which individuals engage with social networking platforms and online communication channels. It encompasses various activities and behaviors related to interacting, sharing content, consuming information, and connecting with others through social media platforms [66]. The five-item measure adopted from the study [72] to measure social media usage. “(1) I read hard news via social media. (2) I repost photos or videos clips on government or politics. (3) I upload photos or videos shot by myself on non-recreational latest events. (4) How much do you trust the political information you encounter on social media platforms? (5) I write blogs on government or politics, such as politics, economics, or international relations.”

## 4 Estimated results

The findings of the internal reliability are summarized in Table 2.

**Table 2:** Reliability and validity test.

Variables	Manifestation	Factor Loading	CR	CA	KMO	Barlett's test		
						ꭓ^2^	Df	Sig
Political Participation (PP)	PP1	0.88	0.88	0.74	0.81	588	2	0.00
	PP2	0.81						
	PP3	0.79						
	PP4	0.85						
	PP5	0.83						
	PP6	0.81						
	PP7	0.79						
Political Education (PE)	PE1	0.71	0.87	0.82	0.77	613	4	0.01
	PE2	0.72						
	PE3	0.81						
	PE4	0.77						
	PE5	0.69						
	PE6	0.75						
	PE7	0.76						
Political Efficacy (PF)	PF1	0.66	0.84	0.83	0.80	746	5	0.00
	PF2	0.69						
	PF3	0.82						
	PF4	0.81						
	PF5	0.73						
	PF6	0.79						
	PF7	0.77						
Social Media Usage (SM)	SM1	0.85	0.85	0.78	0.83	436	3	0.00
	SM2	0.86						
	SM3	0.63						
	SM4	0.70						
	SM5	0.77						

Note: CR = Construct reliability; CA = Cronbach's alpha; KMO = Kaiser–Meyer–Olkin; AVE = Average variance extracted.

The values of CA of all variables are above than 0.7 indicate internal consistency. The convergent validity is measured through CR, Barlett's test, and KMO values. The empirical findings reveal that CR values are more than standard value of 0.8, KMO values higher than acceptable value of 0.7, and significance level is 0.000, qualify for Bartlett's test. The discriminant validity is determined through Pearson's correlation coefficient and the AVE square root of each variable. The results of discriminant validity are presented in Table 3.

**Table 3:** Discriminant validity.

	PP	PE	PF	SM
**PP**	0.88			
**PE**	0.45	0.85		
**PF**	0.62	0.63	0.86	
**SM**	−0.51	−0.42	−0.59	0.85

Note: All values are significant at 1 % while diagonal value is square root of AVE.

The values of correlation coefficients are smaller than square root of AVE depicting that internal correlations between the observed variables are higher than external correlations, so discriminatory validity exists.

### 4.1 Structural Equation Modeling (SEM)

Structural Equation Modeling (SEM) is a statistical technique used to test and estimate causal relationships between variables. It combines aspects of factor analysis and multiple regression analysis to examine complex relationships among variables. The first step in SEM is to specify a theoretical model that represents the relationships between observed variables (manifest variables) and latent variables (unobserved variables). SEM involves the estimation of both the measurement model and the structural model. The measurement model specifies how latent variables are related to their observed indicators. This step involves confirming that the chosen indicators adequately represent the latent constructs. This model specifies the relationships between latent variables in the model. It allows for the estimation of direct and indirect effects between variables, as well as the overall fit of the model to the data. SEM uses techniques such as maximum likelihood estimation to estimate the parameters of the model. The goal is to find the parameter estimates that best fit the observed data while taking into account the measurement error in the indicators. After estimating the model, the fitness of the model is assessed using various fit indices, such as the chi-square statistic, Comparative Fit Index (CFI), Tucker-Lewis Index (TLI), and Root Mean Square Error of Approximation (RMSEA). These indices help determine how well the model fits the observed data.

SEM allows researchers to include control variables in the model to account for potential confounding variables. These variables are included in the model as exogenous variables that can influence both the independent and dependent variables. SEM can be used to test for mediation effects, where the relationship between an independent variable and a dependent variable is mediated by one or more intervening variables. This helps to clarify the direct and indirect effects of the independent variable on the dependent variable.

SEM method is used to test the hypotheses of the study. The findings are presented in Table 4.

**Table 4:** Structural parameter estimate.

Hypotheses	Standardized Path Coefficient	t-values	Confidence interval	Effect Size	Result
PE→ PP	0.416*	5.623	0.38, 0.46	Medium	Yes
SM → PP	0.387*	6.257	0.34, 0.40	Small	Yes
PF → PP	0.484*	8.346	0.42, 0.55	Medium	Yes
PE → SM → PP	0.433*	8.521	0.38, 0.49	Medium	Yes
PE → PF → PP	0.512*	7.254	0.46, 0.59	Large	Yes

x^2^/df = 2.816* NFI = 0.903.

RMSEA = 0.072 CFI = 0.912.

RFI = 0.922 GFI = 0.915.

IFI = 0.931 RMR = 0.053.

Note: * shows significance at 1 %.

The findings of SEM highlight that all estimated paths are positive and significant, and thus all hypothesis are supported. Various goodness of fit indices are utilized to determine the model's fitness. The (x^2^/df) ratio was below 5, and the RMR score was 0.053, while the RMSEA score was 0.072, both below the acceptable threshold of 0.08. Additionally, the GFI, CFI, RFI, NFI, and IFI values were all greater than the recommended values of 0.90.

## 5 Discussion

This study explores the impact of political education, political efficacy, and social media usage on political participation in association with mediating role of social media usage and political efficacy. The main findings of the study reveal that political education has positive relationship with political participation which confirms the hypothesis 1, the value of path coefficient is 0.416 at 1 % level of significance. In China, political education plays a crucial role in shaping citizens' attitudes, behaviors, and understanding of the political system, influencing their level of political participation. However, it's important to note that political education in China is unique due to the country's political structure and emphasis on state-controlled education and ideological indoctrination [35,61,72]. Political education in China is deeply rooted in promoting the ideology of the Communist Party. Citizens are taught the principles of Marxism-Leninism, Mao Zedong Thought, and Xi Jinping Thought. This ideological education aims to instill loyalty to the Party, fostering a sense of duty and participation in Party activities [37,73]. Political education in China emphasizes civic responsibility and patriotism. Citizens are taught to prioritize the collective good over individual interests and to contribute to the nation's development. This emphasis on nationalism encourages participation in state-sanctioned activities and initiatives [39,74]. China's educational curriculum is designed to promote political education and instill Party-approved narratives. Textbooks and educational materials emphasize the achievements of the Party, historical events, and the importance of Party leadership [75,76]. This indoctrination can influence citizens' beliefs and encourage them to participate in activities aligned with the Party's agenda. Moreover, political education in schools and universities aims to socialize the younger generation into active participants in the Party's vision for the country. Youth-focused programs, such as Communist Youth League activities, encourage political participation among students and young adults. In addition, the Chinese government controls political discourse and information flow, including on social media platforms [65]. Political education reinforces the official narratives, influencing citizens' perceptions and encouraging conformity in political participation aligned with government-approved activities. In China, political education serves as a tool for social control and shaping citizens' behaviors and attitudes toward political participation [38,56]. It promotes participation aligned with the Party's objectives, emphasizing loyalty, civic responsibility, and nationalism. However, it's essential to consider that political participation in China operates within a highly controlled and structured political environment, where dissent or activities contrary to state interests are heavily restricted [55,77].

It is argued that political education can lead to increased political knowledge, which is positively associated with political participation. When individuals are more informed about political issues, candidates, and policies, they are engaged in political activities. Political education also enhances individuals' political efficacy, which refers to their belief in their ability to understand and participate in politics. Higher levels of political efficacy are associated with greater political participation, as individuals are more likely to believe that their actions can make a difference. It can help individuals develop civic skills, such as critical thinking, communication, and advocacy skills, which are important for effective political participation. These skills enable individuals to engage more actively in political activities. It often promotes democratic values such as tolerance, respect for diversity, and a commitment to democratic principles and processes. Individuals who have been exposed to such education are more likely to engage in political activities that uphold these values, such as participating in peaceful protests or supporting democratic reforms. Political education can stimulate interest in politics and encourage individuals to become more engaged in political affairs. By providing opportunities for learning and discussion, political education can inspire individuals to take an active role in shaping their communities and societies. Overall, political education has a positive relationship with political participation by increasing political knowledge, enhancing political efficacy, developing civic skills, promoting democratic values, encouraging political interest and engagement, and empowering marginalized groups.

It is also found from empirical analysis that social media usage increases the political participation which confirm the hypothesis 2, the value of path coefficient is 0.387 at 1 % level of significance. Social media platforms like WeChat, Weibo, and others are used to disseminate political information, news, and opinions. This information sharing influences political awareness among citizens [54]. Social media provides a platform for limited civic engagement, allowing discussions on local issues, sharing information about community initiatives, and facilitating some degree of public discourse. This can increase awareness and engagement at a local level [53]. Younger demographics in China are active on social media, using it to share opinions and participate in discussions, albeit within the bounds of government censorship. Platforms like WeChat are used by the Communist Youth League for engagement and mobilization, encouraging youth participation in state-approved activities [52]. It is also worth noting that social media allows for selective mobilization and advocacy within approved boundaries. Certain campaigns or initiatives aligned with government objectives are permitted, encouraging participation in these sanctioned activities [15,78]. Social media enables sharing information about grassroots initiatives, volunteering opportunities, and community activities, facilitating participation in non-political, community-focused endeavors [22]. Government agencies and officials use social media to disseminate official announcements, policies, and directives. This creates a space for citizens to access and engage with government information [51].

The relationship between social media usage and political participation is a topic of considerable debate and research. While some argue that social media can enhance political participation by providing new avenues for communication and mobilization, others suggest that it may contribute to polarization and disengagement. But it can be argued that social media platforms provide users with access to a wide range of information and news sources, including those outside traditional media channels. This increased access to information can help users become more informed about political issues, candidates, and events, which may motivate them to participate in political activities. Social media facilitates political engagement and mobilization by enabling users to connect with like-minded individuals, join online communities, and participate in discussions and debates. This can lead to the formation of online political movements and campaigns that encourage users to take action in the offline world. Social media platforms provide users with a space to express their political views, engage in political dialogue, and debate issues with others. This can help users develop their political identities, clarify their opinions, and become more confident in expressing their views, which may translate into increased political participation. Social media has become an important tool for political campaigning and advocacy, allowing candidates and organizations to reach a large audience quickly and inexpensively. By using social media to promote their platforms and engage with voters, political actors can encourage greater participation in elections and other political activities. Social media enables users to participate in political activities in new and innovative ways, such as signing online petitions, donating to political campaigns, and sharing political content with their networks. These forms of participation may appeal to individuals who are not inclined to engage in traditional forms of political action. Social media is particularly popular among young people, who may be more likely to engage in political activities through these platforms. By reaching out to young voters through social media, political actors can potentially increase youth participation in politics.

The hypothesis 3 is also supported by empirical analysis which reveal that political efficacy increases the political participation in China, the value of path coefficient is 0.484 at 1 % level of significance. Higher political efficacy could lead individuals to believe in the efficacy of participating in state-endorsed political activities [50,76]. This includes joining Party-affiliated organizations, engaging in government-led initiatives, or participating in activities aligned with the state's objectives [48]. Individuals with a higher sense of political efficacy might feel empowered to address and participate in local issues that are within the boundaries of state approval [72]. This could involve participation in neighborhood committees, community-driven initiatives, or public service activities under state guidance [49]. Higher political efficacy leads to increased participation in state-sanctioned platforms like WeChat groups or Communist Party-led events, where individuals feel their participation might have an impact or align with their beliefs in affecting change [46,79]. The Communist Youth League and state-sponsored youth activities encourage participation among younger demographics. Higher political efficacy could motivate youth to engage in these activities, believing in their potential to contribute positively within the state framework [45]. Individuals with higher political efficacy might be more inclined to engage with government-led initiatives or participate in civic activities guided by government channels, believing in the efficacy of these actions to make a difference within the regulated political environment [44]. Despite higher efficacy, individuals in China understand the constraints of political participation within the boundaries set by the government [52]. Hence, higher political efficacy leads to increased participation within these constraints, respecting and aligning with the state's rules and objectives [17,80].

In the context of China, where the political system is characterized by a single-party rule under the Communist Party of China (CPC), the relationship between political efficacy and political participation is complex. The findings of the study reveal that political efficacy increases political participation in China. Higher levels of political efficacy can empower citizens in China to believe that their actions can make a difference in the political process. This sense of empowerment can motivate individuals to participate in political activities, such as voting, attending political meetings, and engaging in advocacy efforts. Moreover, political efficacy is often linked to trust in the political system and government institutions. In China, individuals who have higher levels of political efficacy may be more likely to trust the CPC and its leadership, which can lead to increased participation in activities that support the government's agenda. It is also related to individuals' understanding of political issues and processes. In China, individuals with higher levels of political efficacy may be more informed about government policies and the rationale behind them, which can lead to more active engagement in political activities. In addition, political efficacy is influenced by individuals' perception of their ability to influence political outcomes. In China, individuals who believe that they can influence government decisions may be more likely to participate in political activities to exercise this influence. In China, individuals who have been socialized to believe in their ability to participate in politics may be more likely to engage in political activities. The Chinese government actively promotes political participation through various channels, including state-controlled media, educational institutions, and grassroots organizations. This mobilization can enhance individuals' sense of political efficacy and encourage them to participate in government-sponsored activities. Overall, political efficacy increases political participation by empowering citizens, fostering trust in the political system, enhancing understanding of political issues, promoting a sense of influence, and influencing civic education and socialization.

The findings of the study also depict that social media usage and political efficacy mediates the relationship of political education and political participation which confirm the hypothesis 4 and 5, the value of path coefficient are 0.433, and 0.512 respectively, at 1 % level of significance. Political education enhances individuals' knowledge, understanding, and critical thinking skills about political processes [47,81]. This increased education can positively influence individuals' political efficacy by boosting their confidence in understanding politics and participating effectively [41,48]. Social media serves as a platform for accessing information, engaging in political discussions, and mobilizing individuals for participation. Individuals with higher political education levels might actively seek out diverse viewpoints and engage in political discussions on social media, which could positively influence their political efficacy [40,49].

The relationship between political education, social media usage, and political participation is complex, and social media usage can play a mediating role in this relationship. Social media platforms provide access to a wide range of political information and news sources. Individuals who have received political education may be more proficient at critically evaluating the received information and using it to form political opinions. Social media can facilitate the dissemination of this information to a wider audience, potentially increasing political participation. It can be argued that social media platforms can serve as important tools for political engagement and mobilization. Individuals who are active on social media may use these platforms to organize and participate in political activities, such as online discussions, campaigns, and protests by enabling individuals to connect with like-minded individuals. Social media platforms also facilitate the formation of online communities based on shared political interests and identities. Individuals who are part of these communities may feel a stronger sense of belonging and solidarity, motivating them to participate in collective political activities. Social media serves as a medium through which educated individuals can connect with others who share their political views and values, reinforcing their political education and encouraging political participation. It can be concluded that social media usage mediates the relationship between political education and political participation by facilitating information access and dissemination, enhancing engagement and mobilization, fostering civic skills development, promoting political efficacy and engagement, and facilitating community and identity formation.

Higher political efficacy, influenced by political education, might motivate individuals to engage more actively on social media in political discussions, advocacy, or sharing information about political events. This increased engagement on social media can, in turn, encourage further political participation [47,52]. Political education might equip individuals with critical thinking skills to discern credible information on social media platforms. Educated individuals are more likely to engage critically with political content, contributing to a more informed and participatory online discourse [44]. Individuals with higher political efficacy, stemming from their education and enhanced by social media engagement, are more likely to believe in their capacity to influence political outcomes [15]. This belief can motivate them to actively participate in various political activities, such as voting, attending rallies, or engaging in advocacy efforts [17]. In summary, political education positively influences political efficacy, which, in turn, impacts social media usage and subsequent political participation. The interplay between these factors forms a complex network where higher education levels foster critical engagement on social media, enhancing political efficacy and encouraging greater participation in political activities [28].

Political efficacy also plays a mediating role in the relationship between political education and political participation. As the political education enhances individuals' understanding of political processes, institutions, and issues. This increased understanding can lead to higher levels of political efficacy, as individuals feel more capable of navigating the political landscape and influencing political outcomes. Political education can also boost individuals' confidence in their ability to participate in politics. By providing them with the knowledge and skills necessary for political engagement, political education can increase individuals' self-efficacy in political matters, leading to greater political participation. Higher levels of political efficacy can motivate individuals to participate in political activities. Individuals who feel confident in their ability to understand and influence politics are more likely to engage in activities such as voting, attending political meetings, and engaging in advocacy efforts. Political efficacy can mediate the relationship between political education and political participation by serving as a psychological mechanism that links the two. Political education can enhance political efficacy, which in turn can increase political participation. In this way, political efficacy mediates the relationship between political education and political participation and plays a crucial mediating role in the relationship between political education and political participation by enhancing individuals' understanding, confidence, sense of control, motivation, and ultimately, their willingness to participate in political activities.

### 5.1 Theoretical implication

The findings of the study have several theoretical implications that can deepen our understanding of political education, political participation, social media usage and political efficacy. This study confirms the civic empowerment theory which states that political education, social media engagement, and increased political efficacy collectively contribute to civic empowerment. It suggests that informed citizens who engage critically with political information and platforms are more likely to feel empowered to participate actively in the political process. The findings also support the notion of digital citizenship theory, emphasizing the importance of digital literacy and engagement in modern democratic societies. It highlights that effective utilization of social media as a tool for political discussion and engagement is linked to enhanced political efficacy and subsequent participation. The positive relationships observed in the study align with the principles of participatory democracy. It emphasizes the significance of informed and engaged citizens in influencing political decisions and shaping governance processes through active participation facilitated by political education and social media. Moreover, the relationship underscores the role of social networks and connections formed through social media and political education in fostering social capital theory. It suggests that these factors contribute to the creation of networks that encourage political efficacy and participation, enhancing the overall social capital within a society.

### 5.2 Practical implications

This study offers various practical implications that can guide policy-making, education, and civic engagement initiatives. Emphasize and invest in comprehensive civic education programs that promote critical thinking, information literacy, and civic responsibility. These programs should include components focused on digital citizenship and responsible social media engagement. Integrate digital literacy and responsible social media use into educational curricula at various levels, emphasizing the importance of using social media platforms as tools for civic engagement and informed political discourse. Provide professional development and training for educators to effectively teach digital literacy skills, critical thinking, and the responsible use of social media in the context of civic education. Encourage open and respectful dialogue on social media platforms by supporting initiatives that foster constructive political discussions and diverse viewpoints while combating misinformation and polarization. Support community-based initiatives that leverage social media for raising awareness, engaging citizens in local governance, and fostering grassroots activism. Develop and implement digital literacy programs targeting various demographics, especially youth, to equip them with the skills needed to critically evaluate online information and participate meaningfully in political discussions. Inform policy development on digital rights, access to information, and online discourse, considering the role of social media in fostering political efficacy and participation. Create targeted engagement strategies using social media to encourage youth participation in politics, leveraging their familiarity with digital platforms to promote civic engagement. In light of social media's growing influence, political education programs can be designed or adapted to leverage social media through integration of lessons on digital citizenship, critical media literacy, and online political engagement into political education curricula. Teach students how to critically evaluate information online, identify misinformation, and engage in constructive political discourse on social media platforms. Use social media platforms to disseminate educational materials, share resources, and facilitate discussions. Create interactive online modules, webinars, and live streams to engage students and promote active learning. Encourage students to actively engage with political issues on social media by sharing articles, participating in discussions, and expressing their opinions. This can help students develop their political identity and voice, as well as increase their awareness of current events. Encourage students to participate in digital civic engagement activities, such as online petitions, letter-writing campaigns, and virtual town halls. Teach them how to use social media to amplify their voices and advocate for change on issues they care about. Teach students how to protect their privacy and security online, and how to navigate the digital landscape responsibly. Emphasize the importance of verifying information before sharing it and being mindful of the impact of their online actions. By incorporating these suggestions into political education programs, educators can help prepare students to be informed, engaged, and responsible citizens in an increasingly digital world.

### 5.3 Limitation and future research

This study has also some limitations which may be addressed in future studies. This study has a limited sample which may be extended by incorporating specific socio-economic backgrounds and findings may be generalize to the broader population. Moreover, many other factors may be included in another study, such as cultural influences, historical contexts, or other individual characteristics. These factors may influence the observed relationship of this study. Future studies may incorporate the temporal sequence of studied variables. In addition, the findings of the study might be context-specific and might not be easily applicable to different cultural or political contexts due to variations in political systems, media regulations, or social norms. Recognizing and addressing these limitations in future research can strengthen the validity and applicability of studies exploring the complex relationships between political education, social media usage, political efficacy, and political participation.

The study's sample is limited to residents of China, which may restrict the generalizability of the findings to other cultural or political contexts. Future studies could benefit from a more diverse sample that includes participants from various political systems and cultures to examine if the observed relationships hold in different settings. Future research could incorporate qualitative methods or mixed methods to gain a more nuanced understanding of how political education influences political participation and the role of social media in this process.

## 6 Conclusion

Political education has prime importance and wider implications in context of political participation. There is a literature gap regarding exploration of impact of political education on political participation in China which filled this study. Moreover, the impact of political efficacy and social media usage is also explored along with their mediating role in relationship of political education and political participation. This study collected the primary data from 843 Chinese residents and applied structural equation modelling technique for empirical analysis. The study's findings illuminate a compelling relationship between political education, social media usage, political efficacy, and political participation, underscoring the interconnectedness of these factors in shaping political participation. The positive associations observed among these variables offer insightful implications for fostering informed, active, and engaged citizenship.

Political education emerges as a fundamental pillar in nurturing citizens' understanding of political processes, enhancing critical thinking skills, and fostering a sense of civic responsibility. This education empowers individuals to navigate the complexities of the political landscape, contributing to the development of an informed electorate capable of meaningful participation. Moreover, the study highlights the pivotal role of social media as a catalyst for political engagement. Social media platforms serve as accessible spaces for information dissemination, deliberation, and mobilization, fostering dialogue and connections among diverse populations. Engaging with political content on these platforms not only facilitates knowledge acquisition but also shapes individuals' political efficacy, enhancing their belief in their capacity to effect change. The mediating role of political efficacy emerges as a crucial link between education, social media, and political participation. Individuals with heightened political efficacy are more likely to engage actively in political activities, whether through voting, community involvement, or digital advocacy. This underscores the significance of instilling confidence in citizens regarding their ability to influence political outcomes.

The study's implications extend beyond academia, providing actionable insights for policymakers, educators, and stakeholders invested in cultivating democratic participation. It underscores the need for comprehensive civic education programs that integrate digital literacy and responsible social media usage. Initiatives promoting open dialogue, community engagement, and collaboration among governments, civil society, and social media platforms are essential in fostering a robust civic culture.

## Data availability statement

The data can be gained from authors without any reservation.

## Funding information

2023 Shandong Provincial Social Science Planning School Ideological and Po_x0002_litical Education (Cultivating Virtue and Cultivating People in the Whole Environment) Research Project: Exploration of the Bridging Teaching Mode of Xi Jinping Socialist Thought with Chinese Characteristics in the New Era from the Perspective of Integration, Approval No. 22CSZJ37.

## CRediT authorship contribution statement

**Mo Chen:** Methodology, Investigation, Funding acquisition, Formal analysis, Data curation. **Ghulam Rasool Madni:** Writing – original draft, Methodology, Investigation.

## Declaration of competing interest

The authors declare the following financial interests/personal relationships which may be considered as potential competing interestsMo Chen reports financial support was provided by 2023 Shandong Provincial Social Science Planning School Ideological and Po_x0002_litical Education (Cultivating Virtue and Cultivating People in the Whole Environment) Research Project Approval No. 22CSZJ37. If there are other authors, they declare that they have no known competing financial interests or personal relationships that could have appeared to influence the work reported in this paper.
